# Exploring the effects of mapping rule switching on motor preparation in young and older adults: evidence from combining response cuing and task switching methodology

**DOI:** 10.1007/s00426-025-02150-z

**Published:** 2025-07-08

**Authors:** Jos J Adam, Iring Koch

**Affiliations:** 1 Department of Nutrition and Movement Sciences, Faculty of Health, Medicine and Life Sciences, Maastricht University, P.O. Box 616, Maastricht, 6200 MD The Netherlands; 2 Institute of Psychology, RWTH Aachen University, Jägerstr. 17/19, Aachen, D-52056 Germany

**Keywords:** Proactive control, Motor preparation, Task switching, Working memory, Aging

## Abstract

This study explored the effect of mapping rule switching on motor preparation in young and older adults. Motor preparation was indexed by performance in the finger cuing task, which is a cued 4-choice reaction time (RT) task requiring a single keypress with 1 of 4 fingers (index and middle fingers of both hands). Mapping rule switching required switching between two possible mapping rules implemented via spatially compatible procues and spatially incompatible anticues. These informative cues preceded the target signal at five different time intervals (100–850 ms) to assess the temporal dynamics of preparatory control relative to a non-informative (control) cue. In the single-mapping condition, procues and anticues were administered in separate trial blocks. In the mixed-mapping condition, procues and anticues were randomly intermixed, with a mapping rule cue appearing at trial onset. Analyses of (absolute) RTs and (proportional) cuing effects in single-mapping and mixed-mapping conditions revealed greater preparation benefits for procues than anticues (only at short preparation intervals), and smaller preparation benefits for older than younger adults (only at longer preparation intervals). In both age groups, switching between mapping rules in the mixed-mapping condition created mixing costs (relative to single-mapping), reflecting substantial deficits in motor preparation, and more so at longer preparation intervals where proactive control dominates. These findings reveal a strong impact of mapping rule switching on motor preparation. We propose that activating a new mapping rule and preparing an action both require updating operations in working memory that bias response selection mechanisms.

## Introduction

Advance preparation is remarkably powerful in optimizing performance in many real-life domains (sports, driving, decision-making). At the same time, sudden changes in the environment may instantaneously prioritize a new action goal (braking instead of accelerating), a new mapping rule (respond with the left instead of the right hand), or even an entire new task (answer the phone), all of which require a quick switch in behaviour that may hinder or interfere with advance preparation. Even though well researched in isolation, the link between task switching (for reviews, see Kiesel, Steinhauser, Wendt, Falkenstein, Jost, Philipp, & Koch, 2010; Koch et al., 2018) and action preparation (for reviews, see Jennings & Van der Molen, 2005; Requin et al., 1991) has received little or no attention. In this study, we explored one specific aspect of task switching, namely switching between mapping rules, and how this affects the ability to prepare motor responses in healthy younger and older adults. By integrating experimental tools and theoretical frameworks from two paradigms ─ task switching and motor preparation ─ we aimed to investigate commonalities between the two, thereby exploring new insights that may help in understanding the underlying control principles.

Generally, advance preparation or *proactive* control enhances performance. Hence, usually it trumps *reactive* control, which operates in the absence of reliable prior information, requiring a more direct, but largely unprepared, response to the target signal. According to the dual-mechanisms framework of cognitive control, the dynamic interplay between reactive and proactive control drives flexible, goal-directed behaviour (Braver, 2012; Braver et al., 2021). In this view, proactive control reflects the anticipatory selection and maintenance of goal-relevant information in working memory in order to optimize performance. This control strategy, however, may induce a cost, as it will reduce the capacity for selection (and maintenance) of other meaningful information. Reactive control, on the other hand, reflects transient, stimulus-driven activation of task goals, which imposes smaller demands on working memory.

There is strong evidence that people can make use of advance information to select and prepare motor responses, with improvements in speed and/or accuracy of responding. For instance, studies that employed the finger cuing task (which we describe in more detail below) have reported robust reaction time (RT) and accuracy benefits for cues specifying a left-hand or right-hand response (Miller, 1982). Between-hands preparation is also possible, but it requires longer preparation time (Reeve & Proctor, 1984). Likewise, people can use advance information to prepare a new task set. Studies have shown that cues specifying the to-be-executed task can reduce the costs of task switching when the cuing interval is long enough (Meiran, 1996; see Koch & Kiesel, 2022; Koch et al., 2018, for recent reviews).

In the present study, we examined the relationship between the preparatory processes involved in motor preparation (as studied with the finger cuing paradigm) and cued task switching operationalized in terms of switching between two possible mapping rules (reflecting spatially compatible and spatially incompatible cue-response relationships). As stated before, most studies investigated these preparatory processes in isolation (for a notable exception, see Ruge et al., 2009). Yet, preparing for a specific action and preparing a new task both require proactive control in *updating* the task requirements by selecting, activating and maintaining high-priority task relevant information in working memory, including the current action goal, the relevant mapping rule, and the appropriate effector(s). To explore this common functional basis, we adopted and combined procedures from task switching and response preparation methodology to examine the effect of mapping rule switching on the ability to prepare a subset of finger responses.

Given our specific study aims with respect to the interplay of cognitive control of task set (in task switching) and motor preparation (in finger cuing), we also examined potential age-related performance differences by comparing performance of a young group of participants with that of an older group of healthy participants. We deemed age-related effects to be particularly informative with respect to our exploration of the relationship between task switching and motor preparation because older age affects both motor preparation (Proctor et al., 2005) and task switching (see Chen & Hsieh, 2023; Wasylyshyn et al., 2011, for meta-analyses). Hence, the pattern of age-related effects can inform us about the underlying mechanisms of interactions at the motor level and the task (i.e., mapping rule) level, as we elaborate further below.

In the following, we explain in more detail the response cuing and task switching paradigms, together with some of their key findings, theoretical conceptualizations, and aging effects. We end with the specific aims and hypotheses of the current study.

## Motor preparation: finger cuing paradigm

Miller (1982) developed the finger cuing paradigm, which is a 4-choice RT task that requires participants to respond to spatial-location targets with discrete keypress responses from the index and middle fingers of both hands (placed on a linearly arrayed set of four response keys). At the start of each trial, four possible target locations (empty boxes) appear on the computer screen. Before the onset of the target signal, which indicates the required finger keypress response, a spatial cue signal appears in 2 of the 4 possible target locations. This allows proactive control by selecting and preparing 2 out of 4 possible finger key press responses. If successful, a RT benefit accumulates relative to the control, no preparation, condition. This is because the informative cue reduces stimulus-response uncertainty by half, that is, it transforms the standard 4-choice task into a smaller, less difficult, 2-choice task. To track the time course of preparation, the interval between onset of the cue and onset of the target is varied. Short preparation times, implemented as short cue-target intervals, allow no or minimal preparation and thus generate no or, at best, a small RT benefit. Hence, cognitive control with short preparation intervals is predominantly of the reactive type. In contrast, longer preparation intervals allow more time to select and prepare the cued responses and therefore bring large(r) RT benefits, reflecting successful advance motor preparation.

Unilateral, left-or-right cues in the finger cuing paradigm specify two fingers on one hand. These cues are particularly effective in producing a RT benefit, much more than bilateral cues, which specify two fingers on different hands. According to the grouping model (Adam et al., 2003, 2005), the processing advantage of left-right cues simply reflects the strong grouping of the two leftmost and two rightmost stimulus–response elements. This allows a reflexive shift of attention to the cued side, which in turn leads to a fast, automatic activation (“priming”) of fingers on the same hand (McBride et al., 2012). In contrast, bilateral cues require binding of separate items across the two hands and two hemispheres. Hence, whereas unilateral left–right cues activate responses in a fast, reflexive manner, bilateral cues must activate responses in a slower, effortful way, requiring intentional, top-down cognitive control (Adam & Koch, 2009).

In the present study, we only used left-right cues, which could be compatible (procues) or incompatible (anticues) with the to-be-prepared hand (Adam et al., 2015). Procues represent spatially compatible mappings between cue location and response hand: a left-side cue indicates a left-hand response and a right-side cue indicates a right-hand response. In contrast, anticues employ a reversed, mirror-symmetrical mapping: a left-side cue signals a right hand response, and a right-side cue signals a left hand response. Of course, this cardinal “compatibility” difference between procues and anticues is not without consequences: Due to spatial compatibility, procues produce RT benefits already with short-duration preparation intervals. As argued before, this is because they are driven by a fast, reflexive process of automatic response activation (Adam et al., 2003, 2005, 2015; Eimer, 1995; Hommel, 1998). With longer preparation intervals, slower-acting intentional preparation processes kick in and magnify these early, automatically generated, small RT benefits (Adam et al., 2003, 2005, 2015).

Conversely, if short-duration procues automatically activate ipsilateral responses, it follows that short-duration anticues should produce performance deficits. Indeed, instead of RT benefits, short-duration anticues invariably generate substantial RT *costs* relative to the non-informative control condition (Adam et al., 2015). Again, this happens because anticues automatically activate the ipsilateral, that is, wrong responses, which increases the level of response conflict in the motor system. Overcoming this increased competition requires response inhibition, also called “inhibition for competition resolution” (Duque et al., 2013), which inflates RT (Ridderinkhof et al., 2014).

Long-duration anticues, however, are able to produce RT benefits. This is because they allow sufficient time for proactive control to take charge and to reconfigure the perceptual-motor task set by selecting and activating the correct contralateral fingers. Consequently, with longer preparation intervals, the initial RT cost transforms into a RT benefit, usually equalling that of long-duration procues (Adam et al., 2015). Hence, differential procue and anticue effects are particularly evident at short preparation intervals (i.e., shorter than 450 ms), primarily reflecting differences in reactive control. With longer preparation intervals, slower-acting, top-down preparatory control processes nullify these early occurring, opposing pro/anti cue effects.

What is the effect of older age on preparation performance in the finger cuing paradigm? The first studies on this issue showed stronger age-related deficits in between-hands preparation than in within-hands preparation (Adam, Paas, Teeken, van Loon, van Boxtel, & Jolles, 1998; Adam, Jakob, Bovend`Eerdt, van Gerven, 2012). According to the grouping model, this happens because between-hands preparation more than within-hands preparation relies on age-sensitive proactive control (Adam et al., 2003, 2005). Note, however, that within-hands preparation is not immune against the negative effects of age. Van Gerven and colleagues (2017) showed significant age-related reductions in within-hand cuing benefits, especially at the longer preparation intervals of 450–850 ms, where proactive control dominates. The negative effects of older age on proactive control has also been demonstrated in other paradigms such as the AX-CPT task that assesses updating of context information (Braver et al., 2005). Reactive control, on the other hand, as indexed by performance on the picture-word Stroop task and the Eriksen flanker task, appears to be largely resistant against the negative effects of older age (Bugg, 2014; Verhaeghen, 2011). A recent aging study independently assessed reactive and proactive control in younger and older people and reported unambiguous evidence that older age comes with declines in proactive, but not reactive, control (Ball et al., 2023). This finding parallels evidence documenting a shift from a strong proactive control mode in adult life to a more reactive control mode in older age (Braver et al., 2005; Van Gerven, Hurks, Bovend`Eerdt, & Adam, 2016).

## Cognitive task selection: task switching paradigm

Similar to the cue-based preparation of motor sets (e.g., left or right hand) in the finger cuing paradigm, it is also possible to investigate cue-based preparation at the level of entire task sets using the task switching paradigm (see Koch & Kiesel, 2022; Koch et al., 2018, for recent comprehensive reviews). In cued task switching, each trial (i.e., each target stimulus) is preceded by an explicit task cue. The main finding is that performance (RT, error rates) is worse in task switches than in task repetitions. Notably, there are two different types of such performance costs of task switching. One type of costs can be examined when comparing single-task performance (i.e., AAAA or BBBB) with task switching performance (e.g., ABAB), and the resulting costs have been termed “global” switch costs (Kray & Lindenberger, 2000; Mayr, 2001) or “mixing costs”. The other type of costs is more “local” and is often simply called “switch costs”; this can be examined in mixed-task blocks (e.g., AABB) as the performance difference of task switches vs. repetitions in the same block of trials. It has been found that both global and local switch costs get smaller with long preparation interval, suggesting cue-based advance task-set activation (Kiesel et al., 2010, for a review).^1^

Studies on age-related performance differences in task switching confirmed the typically observed general slowdown of performance in RT tasks, but the more local switch costs are not specifically increased, as indicated by a meta-analysis by Wasylyshyn et al. (2011) and recently confirmed by Chen and Hsieh (2023). In contrast, the more global switch costs or mixing costs (i.e., single-task vs. mixed-task comparison) shows reliable age-specific impairments in proactive mechanisms of maintaining and updating task sets in working memory. Increased global switch costs have also been observed together with a general reduction in the efficiency of preparatory processes in very complex information processing conditions, such as when facing task overlap with bivalent stimuli (Hirsch et al., 2016; see also Mayr, 2001) or when switching between three tasks (Lawo et al., 2012).

In the present study we focused on one isolated aspect of task switching, namely the updating of mapping rules in working memory that specify the spatial relationship between the location of the informative cue (left or right side) and the responding hand (left or right hand) in the finger cuing task. In this constellation, the basic RT task always remains constant and unaltered (4 target locations compatibly mapped to 4 response locations), which deviates from the more typical task switching procedures, which often employ two completely different task sets, each with its own unique stimulus-response sets. The advantage of our approach is that it is easier to attribute possible interactions (between switching and preparation) to mechanisms operating at one specific information processing level. That is, the mapping rule loaded into working memory determines the appropriate responses associated with the motor cue (left hand or right hand responses), thereby biasing a response selection process that chooses 2 from 4 possible responses.

## Aims of study

In this study, we investigated the effects of cued mapping rule switching on motor preparation in the finger cuing task in young and older healthy adults. We examined three issues.

First, we predicted that procue benefits would show up faster (i.e., with shorter preparation intervals) than anticue benefits (relative to non-informative control cues). This would be consistent with previous findings and theoretical considerations (Adam et al., 2015).

Second, we expected age-related impairments in motor preparation (indexed as the proportional cuing benefit, that is, corrected for age-related baseline differences in RT), especially at longer preparation intervals. This follows from the grouping model (Adam et al., 2003, 2005) and dual-mechanisms framework of cognitive control (Braver, 2012), arguing that short preparation intervals evoke transient reactive control, which is largely immune to age-related decline, whereas longer preparation intervals allow slower, effortful proactive control, which is sensitive to age-related decline.

Third, we examined whether mapping rule switching would impair motor preparation. If cued mapping rule switching and motor preparation depend on the same proactive control processes, presumably updating operations in working memory that bias response selection processes, mapping rule switching should hamper motor preparation, especially at longer preparation intervals, where proactive control dominates. This hypothesis would be supported by a smaller preparation benefit in mixed-mapping than in single-mapping blocks, especially at longer preparation intervals.

## Methods

### Participants

Seventy-eight right-handed, healthy individuals with no history of neurological problems were tested from an opportunistic sample. They all were unpaid volunteers, recruited via relatives and acquaintances of third year Biomedical and Health Sciences students at Maastricht University, who collected the data. All participants were free from psychoactive medication and had normal or corrected-to-normal vision. Using a 10-minute cognitive screening tool to detect mild cognitive impairment (Montreal Cognitive Assessment, MoCa; Nasreddine, Bédirian, Charbonneau, Whitehead, Collin, Cummings, & Chertkow, 2005), we removed three older participants from final analysis because they performed below the behavioural cutoff (i.e., they had a MoCA score below 26). In addition, 1 older and 1 younger participant were excluded from final analysis because of poor task performance (i.e., the mean RT was more than three standard deviations above the mean RT of their respective age group). The remaining 73 participants were divided in two age groups: 39 younger adults (age range: 18–26 years; *M =* 20.8 years, 20 male) and 34 older adults (age range: 65–86 years, *M =* 70.8 years, 18 male). The study was approved by the Ethical Committee of the Faculty of Psychology and Neuroscience at Maastricht University. All participants gave written informed consent. Note that a sensitivity analysis using gpower (Faul et al., 2007) showed that, for simple age-related effects, these sample sizes allowed us to detect large to medium effects (d > 0.66) with a power of 0.80.

### Procedure, tasks, and stimuli

**Fig. 1:**
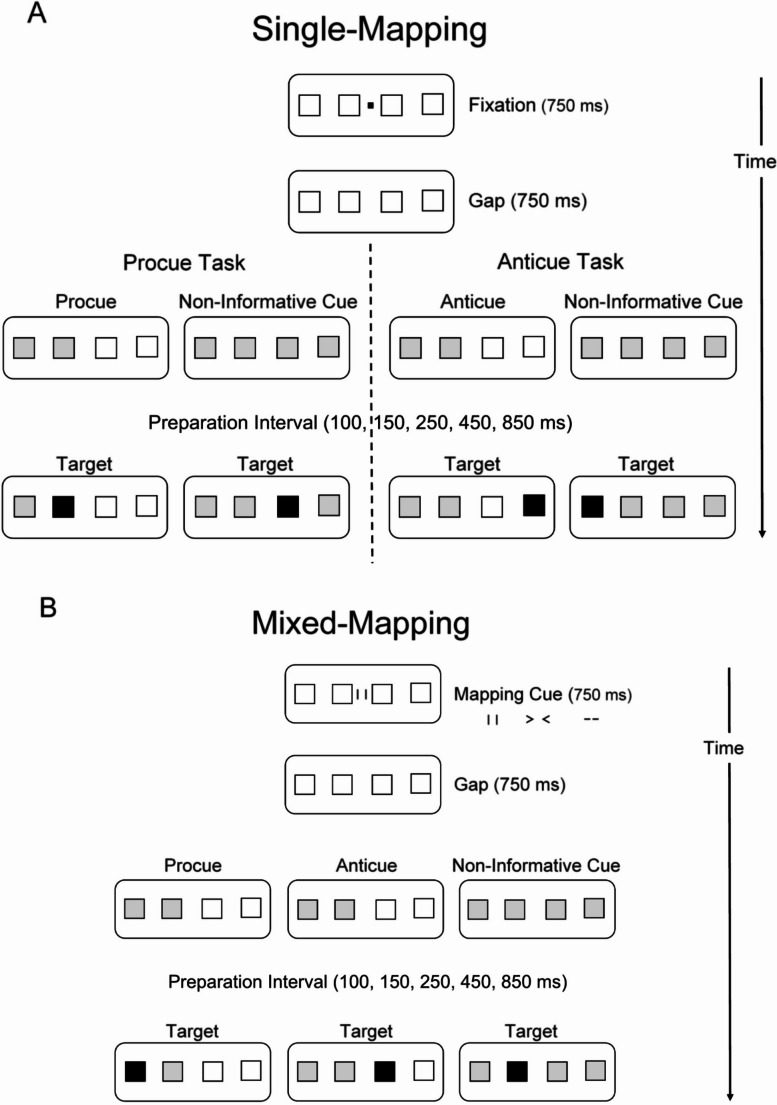
**A** Flowchart of the events in the single-mapping condition that features separate trial blocks for the procue task and anticue task. The participant sees four empty boxes in a horizontal row. After fixation and gap, two boxes (the informative cue) or four boxes (the non-informative cue) turn red (gray in the figure).These cues indicate the target’s possible future locations and associated responses. In the procue task, the informative cue is a spatially compatible *procue*: A left-side cue indicates preparation of the two fingers on the left hand. And a right-side cue indicates preparation of the two fingers on the right hand. In the anticue task, the informative cue is an *anticue*, which indicates the fingers on the opposite side: A left-side cue requires a right-side response and vice versa. Thus, anticues require preparation of the fingers opposite to the side of the cue. In both the procue task and the anticue task there is also a *non-informative* cue, which indicates all four possible response locations; it serves as a control condition against which the effects of the informative procues and anticues can be evaluated. After a random preparation interval of 100, 150, 250, 450, or 850 ms, one box turns green (black in the figure). This is the imperative target stimulus, indicating the required response finger. In the procue, and non-informative cue conditions, the target always appears in one of the cued positions. In the anticue condition, the target always appear in one of the non-cued position. **B** Flowchart of the events in the mixed-mapping condition, where procues, anticues, and non-informative cues are randomly intermixed in one large trial block. The trial starts with a mapping cue that indicates the mapping rule for that trial (note, it replaces the warning signal in the single-mapping condition). The mapping cue symbolizes a procue (||), anticue (> <), or non-informative cue (- -). After mapping cue presentation and gap, two or four boxes turn red (gray in the figure). This is the cue, indicating the target’s possible future locations and associated responses. The *procue *is spatially compatible: A left-side cue indicates a left-side response and a right-side cue indicates a right-side response. The *anticue* indicates preparation of the fingers on the opposite side: A left-side cue requires a right-side response and vice versa. Thus, anticues require preparation of the fingers opposite to the side of the cue. The *non-informative* cue occupies all four boxes, preventing advance preparation of a subset of possible responses. The non-informative condition is a control condition against which the effects of the procues and anticues can be evaluated. After a random preparation interval of 100, 150, 250, 450, or 850 ms, one box turns green (black in the figure). This is the imperative target stimulus, indicating the required finger response

All participants took part in two sessions on separate days. In each session, they performed both the procue and anticue tasks. One day they performed the procue task and anticue task in *separate blocks of trials* that either presented the procues or the anticues, each embedded with non-informative cues; this is the single-mapping condition. See Fig. 1A for a detailed time course of the procue task and the anticue tasks in single-mapping. The other day they performed the two tasks randomly intermixed, that is, they received a mapping cue at the start of each trial indicating the relevant mapping rule (procue, anticue, or non-informative cue): this is the mixed-mapping condition (see Fig. 1B). Hence, in mixed-mapping, mapping rules could change from trial-to-trial, forcing participants to update the relevant mapping rule, thereby creating a “task” switching situation. Order of block type across days and order of task within a day (only for the single-mapping condition) was counterbalanced. A test session on a single day lasted approximately one hour.

#### Procue task (single-mapping condition)

In the procue task, a row of four empty boxes was continuously visible as squares in orange-brown outline on a black background. At the start of each trial, a visual warning signal was presented as a small red square (black in Fig. 1A) midway between the two center boxes. It flickered three times during an interval of 750 ms, after which it disappeared. After an additional waiting period of 750 ms, either all four boxes turned red (neutral cue) or the two leftmost or the two rightmost boxes turned red (procue, gray in Fig. 1A). After a cue-target interval (i.e., preparation interval) of 100, 150, 250, 450, or 850 ms the target signal was presented by making *one* of the cued boxes green (black in Fig. 1A). Note that the preparation intervals increased in a logarithmic way, that is, 50, 100, 200, 400 ms time difference between the successive preparation intervals. Previous work has demonstrated that these intervals nicely capture the temporal dynamics of procue and anticue effects (Adam et al., 2015). Participants were instructed to indicate the location of the target as quickly as possible by pressing the corresponding response key, after which all boxes became empty again. Responses were made by pressing one of four keys on the keyboard (“Z”, “X”, “.”, and “/”; the two leftmost and two rightmost keys on the bottom row). The mapping between stimulus and response key was spatially compatible. Participants operated the response keys with the index and middle fingers of both hands. There was no time limit for responding. When pressing an incorrect response key, an error message was briefly displayed on the screen. The inter-trial interval was 1.5 s.

Participants were informed about the nature of the task and were explicitly told to take advantage of the informative procues. During procue trials, the target always appeared in one of the two cued locations. In trials with non-informative cues, the target appeared in one of the four cued boxes, which precluded selective preparation of a subset of responses.

Five cue-target intervals (100, 150, 250, 450, and 850 ms) were combined with two cue conditions (procue and non-informative), making ten experimental conditions in total. Participants received 20 trials in each of these conditions, totalling 200 trials, which were presented in random order. Short breaks of 30 s separated blocks of 50 trials. Twenty practice trials preceded the test trials.

#### Anticue task (single-mapping condition)

The procedure for the anticue task was identical to that for the procue task, except for the fact that the informative cue was an anticue instead of a procue. That is, in anticue trials, the target signal always appeared in one of the two *non-cued* locations, that is, opposite to the side of the cued locations. Hence, participants were instructed to prepare the fingers on the hand opposite to the side of the cue, that is, the mirror-symmetrical responses (left-side cue → prepare right-hand responses; right-side cue → prepare left-hand responses). Again, there were also non-informative control cues, which did not allow advance preparation of a subset of responses.

#### Procue task and anticue task randomly intermixed (mixed-mapping condition)

In the mixed-task condition, the 200 trials of the procue task (100 procues and 100 non-informative cues) and the 200 trials of the anticue task (100 anticues and 100 non-informative cues) were combined and randomly intermixed, creating 1 large block of 400 trials. Short breaks of 30 s separated blocks of 50 trials, with a longer break of 60 s halfway (after 200 trials). Critically, in the mixed-mapping condition, the visual warning signal (a small red square) that appeared in the single-mapping condition, was replaced by a symbol that indicated the relevant mapping rule: The procue was indicated by two vertical lines “I I” reflecting spatial compatibility. The anticue was represented by two arrows in opposite direction “> <” reflecting the incompatible, mirror-symmetric mapping. The non-informative cue was indicated by two dashes “— —“. These task cues were continuously visible for 750 ms. Forty practice trials preceded the test trials. Otherwise, the procedure was the same as in the single-mapping condition.

## Design

The independent within-groups variables were block type (single-mapping vs. mixed-mapping), task (procue vs. anticue task), cue informativeness (informative vs. non-informative), and preparation interval (100, 150, 250, 450, and 850 ms). Age group (young vs. older) was a between-groups variable. The dependent variables were RT and error rates.

## Analysis

In line with finger cuing methodology, we analyzed absolute RTs in informative and non-informative cue conditions, together with RT cuing benefits/costs for procues and anticues relative to the control, non-informative cue condition (a difference score). Moreover, we analyzed *mixing costs* by comparing cuing effects in single-mapping blocks with that in mixed-mapping blocks.

Please note that the analysis of “local” switch costs, a detailed comparison of cue repetitions and switches in the mixed-mapping condition, was not meaningful due to the presence of non-informative control cues and the manipulation of the preparation time (cue-target interval), which severely limited the number of analysable trials and thus renders this analysis unfeasible.

We removed trials from analyses when the RT was below 200 ms (anticipations) or above 2,000 ms (delayed responses). Then, trials with RTs that deviated more than ± 3 standard deviations from the participant’s overall mean RT were also removed as outliers. Using these outlier criteria, 2.1% of the trials were dismissed. We also removed incorrect trials from the RT analysis, that is, trials with erroneous responses, which occurred on 2.6% of the trials. Of the remaining trials, we calculated mean RT for each participant as a function of Block Type (single-mapping vs. mixed-mapping), Task (procue vs. anticue task), Cue Informativeness (informative vs. non-informative cue), and Preparation Interval (100, 150, 250, 450, and 850 ms).

Mixed analyses of variance (ANOVAs, General Linear Model, SPSS) were performed on RT and error rate. The error rates were arcsine-transformed before analysis to stabilize the variances and reduce skewness of the distribution. We analyzed both untransformed and ln-transformed RTs to correct for the effect of age-related slowing on the RT distribution. Note, we only reported the ln-transformed results if they deviated from the uncorrected analyses. We also performed an ANOVA on the proportional cue effects ((RTnon-informative minus RTinformative)/RTnon-informative). The alpha level was 0.05. We reported effect sizes as partial eta-squared (η_p_^2^) and we applied a Greenhouse-Geisser correction to the degrees of freedom and significance levels whenever the assumption of sphericity was violated (Mauchly’s test).

## Results

We report our findings in three subsections. We start with the findings in the single-mapping blocks, followed by the findings in the mixed-mappings blocks. Then we report a comparison of performance in single-mapping and mixed-mapping blocks to assess global switch costs. Note, again, that the present design, with uninformative cues interspersed in the mixed-blocks, renders a more “traditional” analysis of local switch costs (i.e., switch trials vs. repeat trials in mixed blocks) unfeasible.

### Single-mapping blocks: analyses of motor preparation

#### Reaction time

We performed a mixed four-way ANOVA on RT with Age (young, old) as between-groups factor, Task (procue vs. anticue), Cue Informativeness (informative vs. non-informative), and Preparation Interval (100, 150, 250, 450, and 850 ms) as within-groups factors.

All main effects were significant. Older people produced substantially longer RTs than younger people (555 vs. 371 ms; *F*(1, 71) = 130.13, *p* <.001, η_p_^2^ = 0.65). The procue task yielded shorter RTs than the anticue task (455 vs. 471 ms; *F*(1, 71) = 10.19, *p* <.01, η_p_^2^ = 0.13). Informative cues (i.e., procues and anticues) generated shorter RTs than non-informative cues (457 vs. 470 ms; *F*(1, 71) = 53.82, *p* <.001, η_p_^2^ = 0.43). And RT decreased over the first 450 ms of preparation interval (480, 470, 458, 451, and 456 ms for increasing intervals; *F*(3.18, 225.47) = 55.76, *p* <.001, η_p_^2^ = 0.44). As expected, several significant higher-order interactions qualified these main effects.

**Fig. 2:**
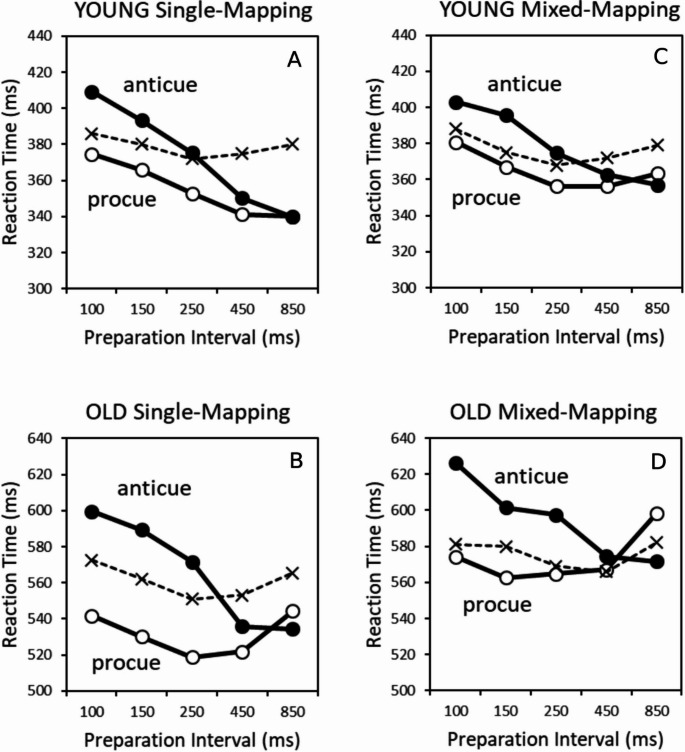
Reaction time in the single-mapping (left row) and mixed-mapping condition (right row) as a function of preparation interval for procues, anticues, and non-informative cues (dashed lines) for younger adults (top row) and older adults (bottom row). Note that in the single-mapping condition, the non-informative cue condition (dashed lines) is displayed as the average RT of the non-informative cue trials in the separate procue and anticue task blocks. Also, note that different (absolute) scales are used for the old and young participants (bottom and top row, respectively), while keeping the RT range identical for both age groups

The significant Task x Cue Informativeness x Preparation Interval interaction, *F*(3.19, 226.31) = 17.61, *p* <.001, η_p_^2^ = 0.20, conformed to our expectations as it reflected a qualitatively different pattern of procue and anticue effects as a function of preparation interval. At short preparation intervals (100–250 ms), procues generated RT benefits relative to the non-informative cue condition, whereas anticues created RT costs. In contrast, at the two longest preparation intervals of 450 and 850 ms, both cue types produced robust RT benefits of similar magnitude. These differential procue and anticue effects were manifest in both age groups as can be seen in Figs. 2A (young adults) and 2B (older adults). These findings support our first hypothesis that procue benefits accrue faster than anticue benefits.

Age modulated the specifics of procue and anticue effects, as suggested by the significant 4-way interaction between Age, Task, Cue Informativeness, and Preparation Interval, *F*(4, 284) = 3.15, *p* <.05, η_p_^2^ = 0.04. Figure 2A and B display this complex interaction. Note that the non-informative cue condition in these figures (dashed line) represents the average RT of the non-informative cue trials performed in the separate procue and anticue task blocks, which were very similar.^2^ Inspection of Figs. 2A, B suggests that older age inflated the opposing effects of procues and anticues at the three shortest preparation intervals. That is, the “spatial incompatibility cost” (the difference between anticue and procue RTs) at the three shortest intervals was much larger for the older than for the younger adults (57 vs. 28 ms, respectively). Similarly, cuing benefits (for procues) and cuing costs (for anticues) relative to the non-informative condition at these three short preparation intervals were in absolute

terms larger for older people (procue benefits: 27 ms; anticue costs: 20 ms) than for younger people (procue benefits: 13 ms; anticue costs: 11 ms). In contrast, at the two longer preparation intervals, there were no differences between procues and anticues in both age groups as they both produced similar preparation benefits. These preparation benefits were smaller in older (procue benefits: 23 ms; anticue benefits: 27 ms) than in younger people (procue benefits: 36 ms; anticue benefits: 33 ms). However, this 4-way interaction did not survive the ln-transformation, *F*(4, 284) = 1.32, *p* =.26, η_p_^2^ = 0.02, so we should be careful in interpreting these absolute RT performance differences.

**Fig. 3:**
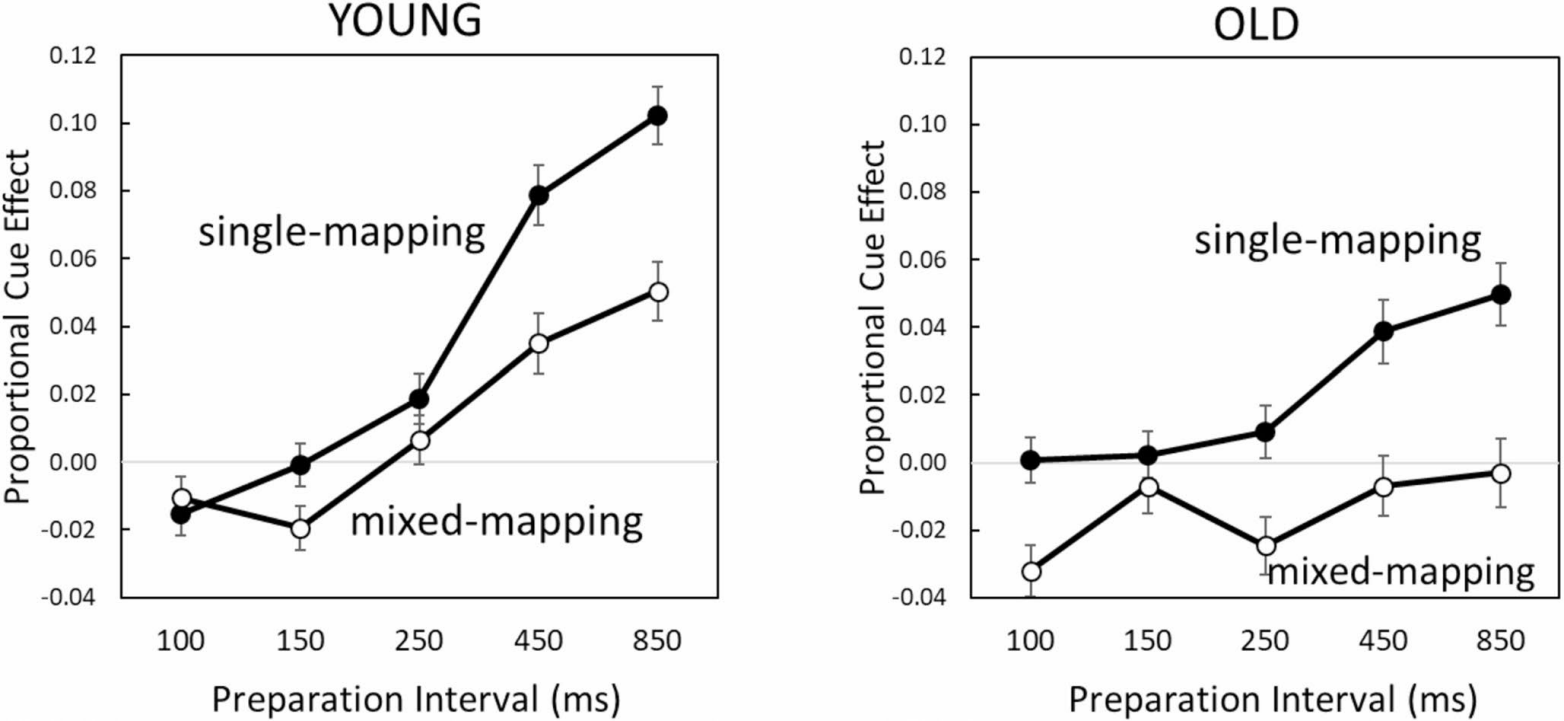
The proportional cue effect ((RTnon-informative – RTinformative)/RTnon-informative)) as a function of preparation interval and block type (single-mapping, mixed-mapping) for younger (left panel) and older (right panel) adults. Overall, preparation efficiency increases with longer preparation intervals. This effect is slower and weaker in mixed-mapping than in single-mapping conditions. Hence, mapping rule switching reduces preparation efficiency and more so with longer preparation intervals. This is the case in both age groups. Overall preparation efficiency is reduced in older compared to younger adults, especially at longer preparation intervals. Error bars denote 1 standard error of the mean

Given the inconsistent findings with raw RT and log-transformed RT scores, we corrected for age-related differences in basic processing speed by calculating a “proportional cuing effect” for each participant in each condition. That is, we derived a difference score (RT non-informative minus RT informative) expressed as a proportion of the baseline RT value provided by the non-informative cue condition in each task ((RTnon-informative – RTinformative)/RTnon-informative). These proportional cuing effects are displayed in Fig. 3, and for the present analysis we focus only on the data of the single-mapping condition (the mixed-mapping blocks are analysed next).

The ANOVA on the proportional cuing effects in the single-mapping condition as a function of Task, Preparation Interval, and Age, revealed a robust effect of Age, *F*(1, 71) = 6.39, *p* <.02, η_p_^2^ = 0.08, reflecting an almost double proportional cuing benefit for younger than older adults (0.037 versus 0.020). Moreover, this overall age effect was qualified by a significant interaction with preparation interval, *F*(4, 284) = 8.46, *p* <.001, η_p_^2^ = 0.11, which indicated that the preparation disadvantage of the older adults only occurred for the two longest preparation intervals of 450 and 850 ms (compare single-mapping for young and older adults in Fig. 3).^3^ This outcome supports our second hypothesis (older age hampers motor preparation especially at the longer preparation intervals), demonstrating that older age interferes in particular with proactive control, leaving reactive control intact.

#### Errors

The overall error rate in the single-mapping condition was 2.5%. Figures.

4 A and 4B display respectively mean error rate for younger and older adults in the single-mapping condition as a function of cue type and preparation interval. We performed a mixed ANOVA on the arcsine-transformed error rates with Age as between-groups factor, and Task, Cue Informativeness, and Preparation Interval as within-groups factors. The significant main effect of Age, *F*(1, 71) = 5.62, *p* <.05, η_p_^2^ = 0.07, revealed fewer errors for older than younger participants (2.1 vs. 3.0%), reflecting a more cautious approach by the seniors. The factor Age interacted with Preparation Interval, *F*(4, 284) = 4.87, *p* <.001, η_p_^2^ = 0.06, demonstrating a stronger decrease of error rates with longer preparation interval for younger than older adults, mostly because younger participants made more errors than older participants at the three shortest intervals (young: 4.0, 3.7, 3.7, 2.1, 1.8%; older: 2.2, 2.5, 1.8, 2.0, 1.7% for increasing intervals).

The significant main effects of Task, *F*(1, 71) = 10.03, *p* <.01, η_p_^2^ = 0.12, and Preparation Interval, *F*(4, 284) = 9.14, *p* <.001, η_p_^2^ = 0.11, mirrored the RT results: Fewer errors in the procue than in the anticue task (2.2 vs. 2.9%) and decreasing error rates with longer preparation intervals (3.1, 3.1, 2.7, 2.0, and 1.7% for longer intervals).

The significant Task x Preparation Interval x Cue Informativeness interaction, *F*(4, 284) = 1.60, *p* <.05, η_p_^2^ = 0.03), indicated that, in the procue task, participants made less errors in the informative than the non-informative cue condition regardless of preparation interval (100 ms: 2.4 vs. 3.6%; 150 ms: 1.5 vs. 3.3%; 250 ms: 1.6 vs. 2.7%; 450 ms: 1.5 vs. 2.2%; 850 ms: 1.0 vs. 2.9%). In the anticue task, a cross-over pattern was manifest, that is, more errors for the informative than the non-informative cue condition at the three shortest preparation intervals and less errors for the informative than the non-informative condition at the longest (850 ms) interval (100 ms: 4.2 vs. 2.4%; 150 ms: 4.8 vs. 2.9%; 250 ms: 4.8 vs. 1.9%; 450 ms: 2.5 vs. 2.0%; 850 ms: 1.1 vs. 2.0%). Again, these findings resemble the RT results.

The significant 2-way Task x Cue Informativeness interaction, *F*(1, 71) = 53.16, *p* <.001, η_p_^2^ = 0.43, indicated in the procue task less errors for informative than non-informative cues (1.6 vs. 2.9%, respectively) but the opposite in the anticue task, more errors for informative than non-informative cues (3.5 vs. 2.3%). This pattern of opposing effects for informative procues (less errors relative to non-informative) and informative anticues (more errors relative to non-informative) was evident in both age groups but more pronounced for younger than older people, as evidenced by the significant three-way interaction Task, Cue Informativeness, and Age, *F*(1, 71) = 5.40, *p* <.05, η_p_^2^ = 0.07, (Young procue: 1.7 vs. 3.5%; Young anticue: 4.3 vs. 2.7; Old procue: 1.5 vs. 2.4%; Old anticue: 2.6 vs. 1.8, for informative and non-informative cues, respectively). This suggests a reduced impact of the informative cues in older age.

### Mixed-mapping blocks: analyses of motor preparation

#### Reaction time

Fig. 2C and D display respectively mean RT for younger and older adults in the mixed-mapping condition as a function of cue type and preparation interval. To ease comparison with performance in single-mapping blocks, we initially performed the same four-way ANOVA as conducted for single-mapping, with Age (young, old) as between-groups factor, Task (procue vs. anticue), Cue Informativeness (informative vs. non-informative), and Preparation Interval (100, 150, 250, 450, and 850 ms) as within-groups factors.^4^

Older adults were slower than younger adults (580 vs. 374 ms; *F*(1, 71) = 103.59, *p* <.001, η_p_^2^ = 0.59). The procue task yielded shorter RTs than the anticue task (473 vs. 481 ms; *F*(1, 71) = 33.40, *p* <.001, η_p_^2^ = 0.32). And RT decreased over the first 450 ms of preparation interval (490, 480, 471, 467, and 476 ms for increasing intervals; *F*(3.38, 239.91) = 25.16, *p* <.001, η_p_^2^ = 0.26). Importantly, and in contrast to the single-mapping condition, the main effect of Cue Informativeness was not significant *F*(1, 71) = 0.81, *p* =.37, η_p_^2^ = 0.01), demonstrating that there was no overall preparation benefit in the mixed-mapping condition (mean RT for non-informative and informative cues were 476 and 478 ms, respectively).

There were two important higher-order interactions, shown in Figs. 2C (young adults) and 2D (older adults). The significant Task x Cue Informativeness x Preparation Interval interaction, *F*(3.54, 251.41) = 22.99, *p* <.001, η_p_^2^ = 0.25, demonstrated that, with short-preparation intervals, procues produced RT benefits whereas anticues produced RT costs (relative to the non-informative control condition). These benefits and costs, however, disappeared with longer intervals.

The significant 4-way interaction with Age, *F*(4, 284) = 3.97, *p* <.01, η_p_^2^ = 0.05, qualified this picture, suggesting that the difference between procues and anticues was inflated in older participants within the three shortest preparation intervals. Moreover, the preparation benefits of procues and anticues, which were manifest in younger adults with the two longest preparation intervals, were basically eliminated in performance of the older adults. Note though that, as in single-mapping, this 4-way interaction did not remain significant when applying the ln-transformation, *F*(4, 284) = 1.89, *p* =.11, η_p_^2^ = 0.03, suggesting a cautious interpretation of this higher-order interaction.

Hence, again, we corrected for age-related differences in basic processing speed by calculating a proportional cuing effect for each participant in each condition. The ANOVA (factors: Age, Task, Preparation Interval) on these proportional cuing effects in mixed-mapping revealed a significant main effect of Age, *F*(1, 71) = 13.47, *p* <.001, η_p_^2^ = 0.16, reflecting a positive overall preparation effect (i.e., a benefit) for younger adults, but a negative overall preparation effect (i.e., a cost) for older adults (0.012 vs. − 0.015). This age effect was qualified by a significant interaction with preparation interval, *F*(4, 284) = 5.75, *p* <.001, η_p_^2^ = 0.08, reflecting that the preparation disadvantage of the older people increased with longer preparation intervals (difference between the two age groups: 0.022, − 0.013, 0.031, 0.042, 0.053 for increasing intervals).^5^ As in single-mapping, this outcome supports the hypothesis that older age impairs motor preparation especially at longer preparation intervals.

In sum, we observed commonalities and differences between mixed- and single-mapping conditions. Later we report the results of a direct statistical comparison of the cuing effects found in single-mapping and mixed-mapping conditions.

#### Errors

**Fig. 4:**
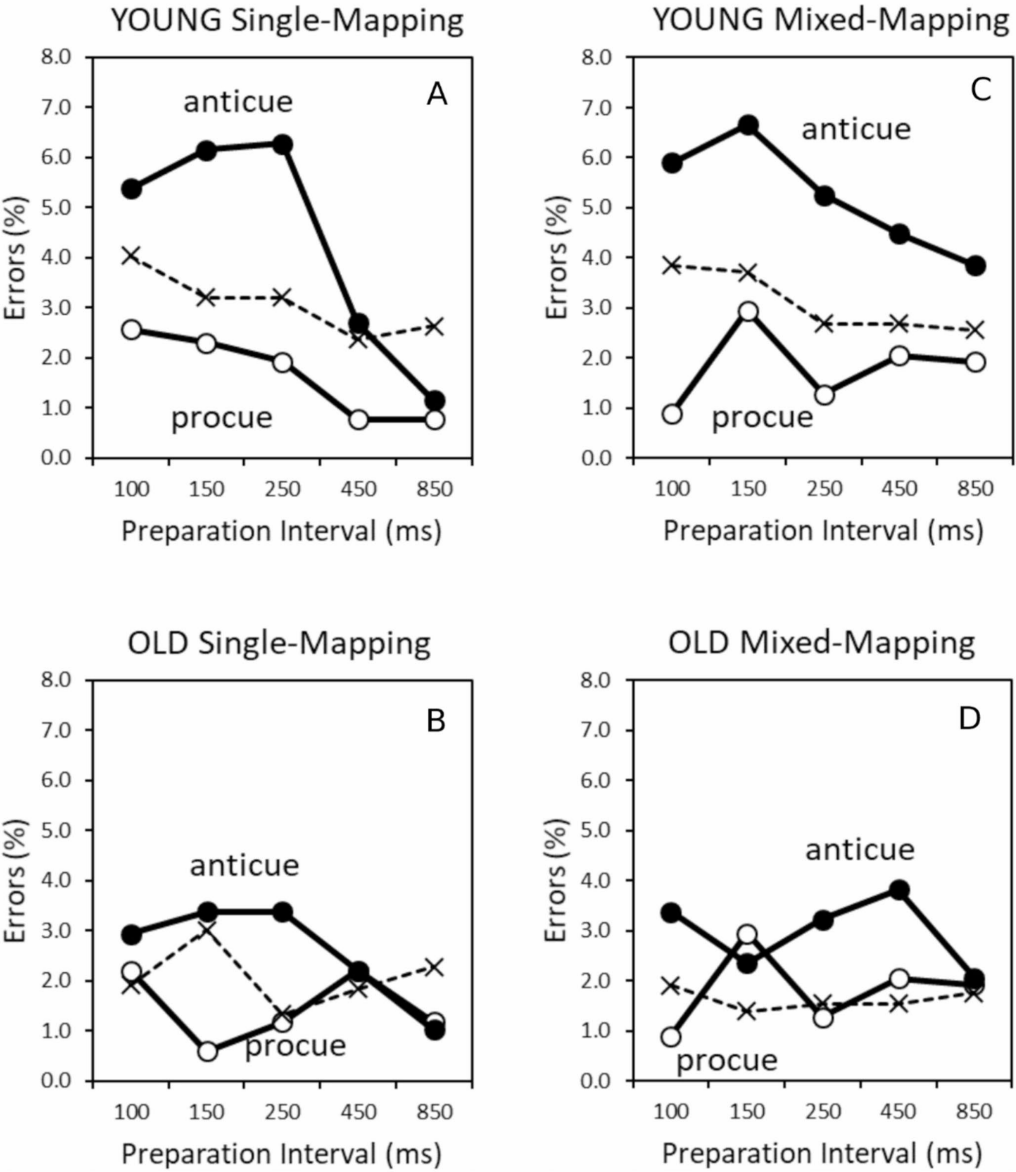
Error rate (%) in the single-mapping (left row) and mixed-mapping condition (right row) as a function of preparation interval for procues, anticues, and non-informative cues (dashed lines) for younger adults (top row) and older adults (bottom row). Note that in the single-mapping condition, the non-informative cue condition (dashed lines) is displayed as the average error rate of the non-informative cue trials in the separate procue and anticue task blocks

The overall error rate in the mixed-mapping condition was 2.6%. Figure 4C and D display respectively mean error rate for younger and older adults in the mixed-mapping condition as a function of cue type and preparation interval. We performed a mixed ANOVA on the arcsine-transformed error rates with Age as between-groups factor, and Task, Cue Informativeness, and Preparation Interval as within-groups factors. The significant main effect of Age, *F*(1, 71) = 8.13, *p* <.01, η_p_^2^ = 0.10, revealed fewer errors for older than younger participants (1.9 vs. 3.3%). The factor Age interacted with Preparation Interval, *F*(4, 284) = 2.43, *p* <.05, η_p_^2^ = 0.03, demonstrating a decrease of error rates with longer preparation interval for younger adults but not for older adults (young: 3.6, 4.3, 3.0, 3.0, 2.7%; older: 2.2, 1.5, 1.8, 2.2, 2.1% for increasing intervals).

The significant main effect of Task, *F*(1, 71) = 68.23, *p* <.001, η_p_^2^ = 0.49, reflected more errors in the anticue task than in the procue task (3.2 vs. 2.0%). The significant interaction between Task and Preparation interval, *F*(4, 284) = 3.87, *p* <.01, η_p_^2^ = 0.05, indicated that the higher error rate in the anticue task decreased with longer preparation intervals (3.8, 3.5, 3.2, 3.1, 2.6% for increasing intervals), but remained low and constant in the procue task (2.1, 2.3, 1.6, 2.0, 2.3% for increasing intervals). The significant Task x Preparation Interval x Cue Informativeness interaction, *F*(4, 284) = 3.87, *p* <.01, η_p_^2^ = 0.05, indicated that, in the procue task, participants made fewer errors with procues than non-informative cues, but only at the three shortest preparation intervals (100 ms: 1.3 vs. 2.9%; 150 ms: 2.0 vs. 2.6%; 250 ms: 1.0 vs. 2.1%; 450 ms: 1.9 vs. 2.1%; 850 ms: 2.3 vs. 2.2%, for procues and non-informative cues, respectively). In the anticue task, participants made more errors with anticues than neutral cues at all preparation intervals (100 ms: 4.6 vs. 2.9%; 150 ms: 4.5 vs. 2.6%; 250 ms: 4.2 vs. 2.1%; 450 ms: 4.2 vs. 2.1%; 850 ms: 3.0 vs. 2.2%, for anticues and non-informative cues, respectively).

Furthermore, the significant 2-way Task x Cue Informativeness interaction, *F*(1, 71) = 68.23, *p* <.001, η_p_^2^ = 0.49, indicated fewer errors with informative (procues) than non-informative cues in the procue task (1.7 vs. 2.4%, respectively) but more errors for informative (anticues) than non-informative cues in the anticue task (4.1 vs. 2.4%).This effect was modulated by Age, *F*(1, 71) = 10.05, *p* <.01, η_p_^2^ = 0.12. Procue benefits and anticue costs relative to the non-informative control cue were robust in young adults (procue: 1.8%; anticue: 5.2%; non-informative: 3.1%), but small or absent in the older adults (procues: 1.6%; anticues: 3.0%; non-informative: 1.6%). As in single-mapping, this suggests a reduced impact of informative cues in older age.

### Comparing single-mapping versus mixed-mapping blocks: mixing costs

#### Reaction time

To directly assess the effect of randomly switching between the procue task (compatible mapping) and the anticue task (incompatible mapping) in mixed-mapping relative to single-mapping, we ran an ANOVA on the proportional cuing effects (again using RT non-informative minus RT informative as a proportion of the baseline RT value indexed by the non-informative condition). The within-groups variables were Block Type (single-mapping, mixed-mapping), Task (procue, anticue), and Preparation Interval (100, 150, 250, 450, and 850 ms). Age was the between-groups factor. In reporting the results of this ANOVA, we focused on the effect of the critical factor *Block Type*, thereby indexing mixing costs. Figure 3 depicts the proportional cuing effects for younger and older adults in single- and mixed-mapping conditions as a function of preparation interval.

The main effect of Block Type was significant, *F*(1, 71) = 45.91, *p* <.001, η_p_^2^ = 0.39, revealing a strong overall negative effect of mapping rule switching on motor preparation (proportional cuing effect: 0.028 vs. − 0.001 for single-mapping and mixed-mapping conditions, respectively). Hence, across age groups, the requirement to switch between procues and anticues on a trial-to-trial basis in mixed-mapping abolished the robust overall preparation benefit that occurred in the single-mapping blocks. Importantly, this effect was modulated by an interaction with preparation Interval, *F*(4, 284) = 6.93, *p* <.001, η_p_^2^ = 0.09, which showed that the negative effect of rule switching on the proportional cuing effect grew with longer preparation intervals (difference between single-mapping and mixed-mapping: 0.013, 0.014, 0.022, 0.045, 0.052 for increasing intervals).^6^ As can be seen in Fig. 3, this was the case in both age groups. These findings corroborate our third hypothesis that mapping rule switching hampers motor preparation especially at the longer preparation intervals. No other effect that included Block Type was significant (all *p* >.1).^7^

The main effect of Age was significant, *F*(1, 71) = 16.08, *p* <.001, η_p_^2^ = 0.19, reflecting an overall much smaller proportional cuing effect in older compared to younger adults (0.003 vs. 0.025). The factor age did not interact with any other variable (all *p* >.1) suggesting that, in the present study, age-related impairments in preparation were not specific to mapping rule switching.

#### Errors

To assess the effect of randomly switching between procues and anticues on response accuracy, we ran an ANOVA on the arcsine-transformed error rates with Block Type (single-mapping vs. mixed-mapping), Task (procue, anticue), Cue Informativeness (informative, non-informative), and Preparation Interval (100, 150, 250, 450, and 850 ms) as within-group factors. Age was the between-group factor. In reporting the results of this ANOVA, we focused on the effect of the critical factor *Block Type*, thereby indexing mixing costs.

The ANOVA results demonstrated three significant effects with Block Type. The significant Block Type x Task interaction, *F*(1, 71) = 8.44, *p* <.001, η_p_^2^ = 0.11, revealed more errors in the anticue than procue task, and rule switching inflated this differential error rate for anticue and procue tasks (single-mapping: 2.9 vs. 2.2%, respectively; mixed-mapping: 3.2 vs. 2.0%, respectively). The significant Block Type x Preparation Interval interaction, *F*(3.346, 237.537) = 3.01, *p* <.05, η_p_^2^ = 0.04, revealed a smaller beneficial impact of longer preparation interval in mixed-mapping than single-mapping (single-mapping: 3.1, 3.1, 2.7, 2.0, 1.7% for longer intervals; mixed-mapping: 2.9, 2.9, 2.4, 2.6, 2.4% for longer intervals). This effect was especially due to a failure to reduce error rate in the informative cue condition with longer preparation intervals in mixed-mapping, as evidenced by a significant 3-way interaction that included cue informativeness, *F*(4, 284) = 2.49, *p* <.05, η_p_^2^ = 0.03. More specifically, in single-mapping, there was a reversal from more errors with informative than non-informative cues at the three shortest intervals to fewer errors with informative than non-informative cues at the two longest preparation intervals (100 ms: 3.0 vs. 3.3%; 150 ms: 3.1 vs. 3.1%; 250 ms: 2.3 vs. 3.2%; 450 ms: 2.1 vs. 2.0%; 850 ms: 2.5 vs. 1.0%, for non-informative vs. informative conditions, respectively). In mixed-mapping, there was no such reversal as there were more errors with informative cues relative to non-informative cues at all preparation intervals (100 ms: 2.9 vs. 3.0%; 150 ms: 2.6 vs. 3.2%; 250 ms: 2.1 vs. 2.6%; 450 ms: 2.1 vs. 3.0%; 850 ms: 2.2 vs. 2.6%, for non-informative vs. informative conditions, respectively). These outcomes suggest a performance decrement in the mixed-mapping condition especially for the informative cues at the two longest preparation intervals, mirroring the RT results. We did not find a single significant effect that involved the factor Age, all *F*s < 1.

## Discussion

In this study, we examined the effect of mapping rule switching on motor preparation in a group of healthy young and older adults. Using a novel approach combining two hitherto kept separate experimental paradigms, we adapted the finger cuing paradigm to include critical elements of task switching. Specifically, using compatible procues and incompatible anticues (together with uninformative control cues), we asked participants to perform the finger cuing task in single-mapping and mixed–mapping conditions and varied the preparation interval to separate reactive control (dominant at short intervals, i.e., shorter than 450 ms) from proactive control (dominant at longer intervals). In the single-mapping condition, the mapping rule was constant by presenting pro- and anti-cues in separate blocks of trials. In the mixed-mapping (rule switching) condition, pro- and anti-cues were randomly intermixed, requiring an explicit mapping-cue at the start of each trial to convey the relevant mapping rule. There were several key findings.

In both age groups, and in both mapping conditions, we found an overall advantage for procues over anticues (both in terms of RT and response accuracy). This procue advantage was strong and robust at the three shortest preparation intervals (100, 150 and 250 ms), but disappeared at the two longest preparation intervals (450 and 850 ms). These results replicate previous findings, demonstrating that procue benefits arise faster than anticue benefits, and, moreover, that late operating intentional preparation processes can override (in case of anticues) or augment (in case of procues) early occurring, autonomous preparatory processes driven by salient left-right cues (Adam et al., 2003, 2005, 2015).

In both mapping conditions, older age reduced (absolute and proportional) cue-driven RT benefits at the two longest preparation intervals (450 and 850 ms), not at the three shortest intervals (100, 150, 250 ms). This finding implicates an empirical dissociation between motor preparation with short and longer preparation intervals, thereby supporting the reactive – proactive control distinction emphasized by the dual-mechanisms framework (Braver, 2012). Moreover, this finding corroborates previous reports of age-related performance decrements in proactive, but not reactive, control (Ball et al., 2023).

Interestingly, aging effects were not significantly different for procues and anticues.^8^ Assuming that inhibitory processes are needed more in anticue than procue conditions (especially at the shortest preparation intervals where they act to combat response conflict) this finding does not support the inhibitory-deficit hypothesis of older age (Lustig et al., 2007). Indeed, a recent meta-analysis of this hypothesis called into question the claim of a general inhibition deficit in older age, arguing that only in a few tasks (go/no-go and stop-signal tasks) older adults show impaired inhibition (Rey-Mermet & Gade, 2018). On the other hand, the present demonstration of an age-related deficiency in motor preparation fits with robust evidence that older age impedes response selection processes in response cuing and stimulus-response compatibility paradigms (Proctor et al., 2005). Hence, the present findings are more consistent with accounts that posit process-specific age-related limitations (West, 1996) than with accounts that claim a general age-related deficit in processing speed (Salthouse, 1996).

Importantly, the absolute and proportional motor preparation benefits observed in the mixed-mapping condition were greatly reduced relative to single-mapping, and most strongly so for the longer preparation intervals, where proactive control dominates. This novel finding suggests a link between mapping rule switching and motor preparation, implying that establishing abstract task rules (“rule activation”) and readying a subset of finger responses (“rule implementation”) are closely related and may draw, at least in part, upon the same processes. One possible source may be the capacity-limited working memory system that, according to the executive-attention theory of working memory capacity, is a strong modulator of proactive control ability (Engle, 2002; Redick, 2014; Redick & Engle, 2011). In this view, cued mapping rule switching in the present study required the capacity-limited working memory system in order to update and maintain the relevant mapping rule, thereby effectively reducing the capacity available for subsequent selective motor preparation, leading to impaired finger cuing performance. In particular, we propose that response selection mechanisms operating in working memory moderate the interacting effects of mapping rule switching and motor preparation. This suggestion is in line with the *response discrimination account* of the Simon effect, the phenomenon of faster and more accurate responses when *irrelevant* stimulus location and response location match than when they do not. According to the response discrimination account, the Simon effect arises from interactions between stimulus-location codes and flexible response representations in working memory rather than from strong stimulus-response associations in long-term memory (Ansorge & Wühr, 2004; Wühr & Ansorge, 2007). Hence, the capacity and content of working memory moderates response selection efficiency and constitutes the primary locus of spatial stimulus-response compatibility effects (Wühr & Biebl, 2011).^9^

As older age reduces working memory capacity (Hedden & Gabrieli, 2004), one might expect older age to inflate the negative impact of mapping rule switching on motor preparation. Indeed, we found a greater difference between the proportional cuing effects established in single-mapping and mixed-mapping conditions for older than younger participants (0.035 and 0.025, respectively), but this age-related difference was statistically not reliable (*p* >.2). Two issues are relevant when interpreting this finding.

First, statistical power. Our sensitivity analysis suggested that we could only detect medium to large age-related effects, suggesting that the effect may have gone undetected if it was actually small. Moreover, our primary outcome measure was the proportional cuing effect, which is a difference score (corrected for overall speed) that has been criticized for use in extreme-groups research because it increases error variance (Draheim et al., 2022).

Second, the current mapping rule switching procedure posed relatively modest demands on working memory capacity, as it required a switching of mapping rules (between cue side and response side) in the context of the same basic stimulus-response set configuration, rather than a switching of completely different task sets, each with its own unique stimulus-response set. Indeed, task switching may take many different forms and it may operate at distinct levels involving different component processes, some of which may be age-insensitive (Meiran et al., 2001). Moreover, in both single-mapping and mixed-mapping conditions, 50% of the trials contained a non-informative control cue, requiring no updating or biasing of working memory content, which may have diluted the “switching-load-contrast”.

As expected, the negative effect of mapping rule switching was most pronounced with the two longest preparation intervals of (450 and 850 ms), where proactive control dominates. Interestingly, we also found small but significant effects within the 150–250 ms preparation interval range. This may suggest a minor impact of mapping rule switching on reactive motor control. Alternatively, it also may be the case that at these shorter preparation intervals proactive control may have started to become (partially) active, only to reach full capacity at longer intervals. This latter possibility fits with the idea that reactive and proactive control process are at least partially independent and thus may overlap and operate in parallel (Braver, 2012).

There are two possible limitations of our study. The first is that we designed our study in a way that we could analyse motor preparation based on cues in the single and mixed blocks by including uninformative cues. This way we could compare the time-course of the informative procues and anticues across the preparation intervals, but this also reduced severely the number of trials that represent direct transitions from procue to anticue trials (or vice versa), so that we could not examine local switch costs. We deemed this inability to assess “local” switch costs (i.e., switch vs. repeat in mixed blocks) as acceptable given that the above-mentioned meta-analysis (Wasylyshyn et al., 2011; see also Chen & Hsieh, 2023, for an updated meta-analysis) did not find consistent evidence for the existence of age-related switch costs. Yet, it would still be interesting to examine age-related effects in local mapping switch costs in future studies.

Another possible limitation of the present study refers to the duration of the mapping cue in the mixed-mapping condition that indicated the relevant mapping rule at the start of each trial. This mapping cue was visible for a constant period of 750 ms. Then it was replaced by a blank interval of 750 ms, after which the left-right motor preparatory cue appeared. Effectively, this procedure created a time window of 1500 ms to encode and consolidate the relevant mapping rule in working memory. Since estimates of working memory consolidation vary between 25 ms and 1000 ms (Carlos, Santacroce, TamberRosenau, 2023), it seems unlikely that the present switch costs were the result of insufficient consolidation of the mapping rule in working memory. On the other hand, the possibilities of temporal decay and attentional refreshing of items that are no longer physically present (Camos et al., 2018) remain an open issue and could be addressed in future work by manipulating the temporal dynamics of the mapping rule cue.

In addition to the present left-right (unilateral) cues, future work could also investigate the effects of mapping rule switching on preparation efficiency with bilateral cues, that is, cues specifying fingers on different hands. Experimental evidence and theoretical considerations have established that bilateral cues are more difficult than unilateral cues, requiring more effortful processing to generate beneficial preparatory effects (Adam et al., 2003, 2005). Inclusion of bilateral cues would allow a strong test of the suggestion that greater processing demands may reveal a robust interaction between aging and mapping rule switching on motor preparation.

In conclusion, the study combined two established experimental paradigms, finger cuing and task switching, in a novel way. The findings revealed that mapping rule switching impaired cue-based motor preparation, especially with longer preparation intervals, where proactive control prevails. This interaction between mapping rule switching and motor preparation suggests a common functional basis, which we tentatively identify as the capacity of working memory. In particular, in line with executive-attention theory of working memory capacity (Redick & Engle, 2011), we propose that response selection processes operating in working memory underlie the interacting effects of mapping rule switching and motor preparation.

## Data Availability

Data are available upon request from the corresponding author

## Footnotes

1 Often “mixing costs” compare performance in single-task conditions, which are by definition task-repetition trials, with that in repetition trials in mixed-task blocks. However, in the present study, we compare it with performance in mixed blocks collapsed across both switch and repetition trials. This is because we included neutral (non-informative) cues in the design, which led to an insufficient number of relevant trials (see Analysis section). The comparison of single vs. mixed blocks has been done in other studies on cognitive aging (e.g., Kray & Lindenberger, 2000).

2 Two-way ANOVAs conducted on the RTs of the non-informative cues in the separate procue and anticue task blocks as a function of preparation interval yielded non-significant terms for the factor task (young: *p* >.25; old: *p* >.24).

3 Independent-samples *T*-tests that examined the significance of the age differences at each preparation interval revealed *p*-values of 0.085, 0.741, 0.374, 0.001, and 0.001 for increasing preparation intervals (2-tailed).

4 In mixed-mapping, the RTs of the non-informative control condition were identical for the procue task and anticue task as procues, anticues, and non-informative control cues were all randomly intermixed in one large trial block. This approach may violate assumptions of the ANOVA statistical model and hence significance values should be viewed with caution. However, we believe this is acceptable at this point in our data analysis. First, in addition to absolute RT, later in this section, we also analyzed the proportional *cuing benefit* (a difference score with the non-informative cue condition as baseline). This eliminates the factor cue informativeness from the ANOVA. Second, and most importantly, in our last data analysis section, we provided a direct statistical comparison of single-mapping vs. mixed-mapping (mixing costs). Here, again we used the *cuing effect* as the dependent variable, which, as stated before, removes the factor “cue informativeness” from the ANOVA, and it is this analysis that provides the critical statistical evidence for our claims regarding the effect of block type (single-mapping vs. mixed-mapping).

5 Independent-samples *T*-tests that examined the significance of the age differences at each preparation interval revealed *p*-values of 0.042, 0.267, 0.010, 0.001, and 0.001 for increasing preparation intervals (2-tailed).

6 Paired-samples *T*-tests that examined the significance of these differences revealed *p*-values of 0.089, 0.041, 0.003, 0.001, and 0.001 for increasing preparation intervals (2-tailed).

7 The mapping rule switching deficit in proportional cuing effects was not due to absolute RT changes in the non-informative (baseline) condition in single-mapping vs. mixed-mapping conditions, *F*(1, 71) < 1, *p* =.38, η_p_^2^ = 0.01. Rather, it was due to absolute RT changes in the informative “preparatory” cue conditions that suffered significantly in mixed-mapping relative to single-mapping, *F*(1, 71) = 8.18, *p* <.01, η_p_^2^ = 0.10.

8 This holds for the proportional cuing effect. Considering absolute RT, older age seems to inflate the RT difference between procues and anticues, especially at shorter preparation intervals (in both mapping conditions, see Fig. 2). In contrast, in terms of error rate, older age seems to decrease the difference between procues and anticues, especially at shorter preparation intervals (see Fig. 4). This opposite set of findings suggests the possibility of a speed-accuracy trade off. Note, however, that this observation has no impact on our main conclusions (less motor preparation in mixed-mapping than in single-mapping; and less motor preparation by older participants than younger ones, especially at longer preparation intervals), which was independent of cue type (procue vs. anticue).

9 An alternative account of the reduced preparation benefits in mixed-mapping relative to single-mapping holds that participants refrained all together from using the preparatory cues in the mixed-mapping condition, possibly because the processing two informative cues is too challenging. This account seems possible, especially for the older adults, but not for the younger adults, as the younger participants showed positive preparation benefits with longer preparation intervals in mixed-mapping (see Fig. 2). Moreover, the alternative account raises the question of why (older) participants would adopt such a strict strategy of ignoring the cues (in the face of explicit instructions to use them). One possibility, in line with our account, is that the imposed processing load may exceed the capacity of working memory.
